# High prevalence and mortality due to *Histoplasma capsulatum* in the Brazilian Amazon: An autopsy study

**DOI:** 10.1371/journal.pntd.0009286

**Published:** 2021-04-05

**Authors:** Natalia Rakislova, Juan Carlos Hurtado, Antonio E. M. Palhares, Luiz Ferreira, Monique Freire, Marcus Lacerda, Wuelton Monteiro, Mireia Navarro, Isaac Casas, Marcus de Melo Teixeira, Paola Castillo, Maria Teresa Rodrigo-Calvo, Lorena Marimon, José Guerrero, Rosauro Varo, Vima Delgado, Llorenç Quintó, Francesc Marco, Emilio Letang, Jordi Vila, Quique Bassat, Clara Menéndez, Jaume Ordi, Miguel J. Martínez

**Affiliations:** 1 ISGlobal, Hospital Clínic, Universitat de Barcelona, Barcelona, Spain; 2 Department of Pathology, Hospital Clinic of Barcelona, Universitat de Barcelona, Barcelona, Spain; 3 Department of Microbiology, Hospital Clinic of Barcelona, Universitat de Barcelona, Barcelona, Spain; 4 Universidade do Estado do Amazonas, Manaus, Brazil; 5 Fundação de Medicina Tropical Doutor Heitor Viera Dourado, Manaus, Brazil; 6 Fundação Centro de Controle de Oncologia do Amazonas, Manaus, Brazil; 7 Instituto de Pesquisas Leônidas & Maria Deane, Fiocruz, Manaus, Brazil; 8 The Pathogen and Microbiome Institute, Northern Arizona University, Flagstaff, Arizona, United States of America; 9 Núcleo de Medicina Tropical, Faculdade de Medicina, Universidade de Brasília, Brasilia, Brazil; 10 Centro de Investigação em Saúde de Manhiça, Maputo, Mozambique; 11 ICREA, Catalan Institution for Research and Advanced Studies, Barcelona, Spain; 12 Pediatric Infectious Diseases Unit, Pediatrics Department, Hospital Sant Joan de Déu (University of Barcelona), Barcelona, Spain; 13 Consorcio de Investigación Biomédica en Red de Epidemiología y Salud Pública (CIBERESP), Madrid, Spain; National Institute for Communicable Diseases, Johannesburg, South Africa, SOUTH AFRICA

## Abstract

**Background:**

Histoplasmosis is acquired by inhalation of spores of the dimorphic fungus *Histoplasma spp*. Although this pathogen is distributed worldwide, it is more prevalent in the Americas. However, the real burden of histoplasmosis remains undefined in many endemic regions.

**Methodology:**

We conducted a series of 61 autopsies to individuals who died in a hospital in the Brazilian Amazon focused on infectious diseases. We performed a detailed histological and microbiological evaluation with genetic characterization of *Histoplasma* strains with the aim to evaluate the contribution of histoplasmosis to morbidity and mortality. Additionally, we assessed the clinicopathological correlation.

**Principal findings:**

Evidence of *Histoplasma* infection was detected in 21 patients (34%). Eight cases were disseminated infections, all of them occurred in HIV-positive patients. Six cases were localized histoplasmosis, limited to the lungs. In seven patients *Histoplasma* DNA was detected by PCR in patients with no histological lesions. *Histoplasma* infection was detected in 38% of HIV-positive patients and was a major contributor to death in 22% of them. Lungs, liver and spleen were affected in all cases of disseminated histoplasmosis. Phylogenetic analysis of the strains suggested a high diversity of *Histoplasma* species circulating in the Brazilian Amazon. Histoplasmosis was clinically missed in 75% of the disseminated infections.

**Conclusions:**

The high incidence of histoplasmosis, the low index of clinical suspicion, and the severity of the disseminated disease highlight the need of proactively implementing sensitive routine screening methods for this pathogen in endemic areas. Antifungal prophylaxis against *Histoplasma* should be encouraged in the severely immunocompromised HIV patients in these areas. In conclusion, substantial mortality is associated with disseminated histoplasmosis among HIV-positive patients in the Brazilian Amazon.

## Author summary

Histoplasmosis is a fungal infection caused by inhalation of spores of the fungus *Histoplasma spp*. It occurs in specific endemic areas, such as areas of USA, Africa and Latin America. However, the real burden of histoplasmosis remains unknown in many endemic regions. Clinically, histoplasmosis is frequently misdiagnosed as tuberculosis. The current study was carried out to explore the frequency and characteristics of *Histoplasma* infection in a series of autopsies conducted in the Brazilian Amazon. We found evidence of *Histoplasma* infection in one-third of the deceased patients. A significant proportion of the cases were disseminated infections, with extensive involvement and severe damage of most organs. All these disseminated infections occurred in HIV-positive patients. Strikingly, histoplasmosis was clinically missed in more than two-thirds of these patients and had an extremely high mortality. In conclusion, the high frequency and mortality of histoplasmosis, together with the frequent misdiagnosis of the disease, highlight the need of implementation of sensitive screening methods for *Histoplasma* in HIV patients in endemic areas. Additionally, antifungal prophylaxis against *Histoplasma* should be encouraged in the severely immunocompromised HIV patients in these areas.

## Introduction

Histoplasmosis is a fungal infection acquired by inhalation of microscopic spores of the dimorphic fungus *Histoplasma spp*. Although this pathogen has a worldwide distribution, histoplasmosis is more prevalent in the Americas [1–4]. In Brazil, histoplasmosis is highly endemic in the Northern regions, particularly in the Amazon, as well as in Mid-Western and South-Eastern areas [4, 5].

Two distinct varieties *Histoplasma* are recognized: *H*. *capsulatum* variety *capsulatum*, responsible for American histoplasmosis, and *H*. *capsulatum* variety *duboisii*, causing African histoplasmosis [3]. Recent genetic analyses have refined the previous taxonomic categorization of the *capsulatum* variety: *H*. *mississippiensis* (NAm1) and *H*. *ohiensis* (NAm2) are endemic to North America while *H*. *capsulatum* (Panama), *H*. *suramericanum* (LAmA) and other genetic clusters such as LAmB, Northeast BR1 and Northeast BR2 are endemic to Latin America and frequently detected in people living with HIV [6].

*H*. *capsulatum* infections in immunocompetent individuals are typically asymptomatic or are associated with mild symptoms, but can cause severe disease in patients exposed to a large inoculum of the fungus [7]. In contrast, immunocompromised patients, especially HIV-positive individuals, are much more likely to develop a disseminated infection [8]. In these patients, disseminated histoplasmosis progresses rapidly and is always fatal if untreated [8]. Unfortunately, despite the scale-up of anti-retroviral therapy, disseminated histoplasmosis is still an important cause of mortality in HIV-positive people living in endemic areas [9]. In Latin America, recent estimations suggest that the incidence and mortality of histoplasmosis in HIV-positive patients may be even higher than those of tuberculosis [10, 11]. However, the real burden of histoplasmosis and its associated mortality in many endemic regions, including Brazil, is poorly known [11, 12].

Most of the clinicopathological data on histoplasmosis is based on case series [13–16] and individual case reports, mostly in HIV-positive patients [17]. The majority of these reports show that lungs, spleen, liver and bone marrow are the most frequently affected organs. However, any tissue can be involved, including central nervous system, adrenal glands, gastrointestinal tract and lymph nodes [18–20]. Clinically, histoplasmosis is frequently missed, partly due to the overlap of its symptoms with those of tuberculosis [1]. However, autopsy studies of fatal histoplasmosis focusing on the clinicopathological correlation are very scarce [17–19, 21, 22].

In this study, we explored the prevalence and mortality associated with histoplasmosis in a series of 61 complete autopsies conducted in a referral hospital located in the Brazilian Amazon [23]. We analyzed the clinical signs and symptoms, the treatments received, and the histopathological and microbiological findings of all cases of histoplasmosis.

## Methods

### Ethics statement

The study received the approval of the Clinical Research Ethics Committee of the Hospital Clinic of Barcelona (File 2013/8677) and the local (Manaus, Amazonas state) and National (Brazil) Bioethics Committees (CAAE 28905514.0.0000.0005/2014). Written informal consent was obtained from the relatives of the deceased individuals.

### Study setting

This study was part of an observational study conducted at the *Fundação de Medicina Tropical Dr*. *Heitor Vieira Dourado* [23], a tertiary referral hospital specialized in the diagnosis and treatment of tropical diseases. The hospital is located in the municipality of Manaus (North East of the Amazonian State). The city population is of 2,094,301 inhabitants, the majority residing in urban and peri-urban areas [24].

### Patients included in the study

We included in this study patients who died at the *Fundação de Medicina Tropical Dr*. *Heitor Vieira Dourado* from March 2014 to February 2015 and fulfilled the following inclusion criteria: (1) a complete autopsy requested by the clinician as part of the medical evaluation of the patient, (2) written informed consent to perform the autopsy given by the relatives and (3) a post-mortem interval time of less than 48 hours.

### Autopsy procedures

Complete postmortem procedure was conducted by a study pathologist with the assistance of a trained pathology technician. Before the procedure, the pathologist thoroughly revised the clinical records of the deceased patient. The autopsy procedures and detailed pathological methods used have been reported elsewhere [25–27]. In brief, the protocol started with the external examination of the body, followed by disinfection of its surface and collection of 20 mL of blood and cerebrospinal fluid for microbiological testing. Afterwards, all the thoraco-abdominal organs, as well as the central nervous system were eviscerated and dissected for detailed gross examination. Then, samples from lungs, liver, spleen, central nervous system and bone marrow were obtained for microbiology and samples of these organs as well as heart, kidneys, stomach, bowel, pancreas, adrenal glands, bladder, lymph nodes, skin, and of uterus in all women of reproductive age, were obtained for pathological evaluation. In addition, any other grossly identified lesion was collected for histological and microbiological analysis. Histological samples were fixed in formalin. Microbiological tissue samples were collected into tubes filled with lysis buffer (ATL buffer, Qiagen, Hilden, Germany).

### Microbiological and pathological methods and cause of death attribution

The general microbiological [25–27] and pathological methods [25, 26] have been reported elsewhere. The study comprised in all cases detection of antibodies against HIV-1/2, polymerase chain reaction (PCR) analyses for respiratory viruses and bacteria, bacterial/fungal culture of blood, cerebrospinal fluid, liver, lungs, and central nervous system. In patients confirmed to be HIV-positive, viral load testing and an additional microbiological screening for common opportunistic pathogens was conducted. Other microorganisms were further explored depending on the specific pathological findings observed.

The histological evaluation included hematoxylin and eosin stain in all samples and histochemical and/or immunohistochemical stains whenever required to reach a cause of death diagnosis.

Once all the analyses of the autopsy samples were completed, a panel composed of a pathologist, a microbiologist, and a clinician with expertise in infectious diseases evaluated all the data of the complete autopsy and the clinical records, and assigned the main diagnosis of cause of death and contributing to death conditions. All conditions involved in the chain of events leading to death were coded following the International Classification of Diseases, tenth revision (ICD-10) [28]. Fundamental diseases contributing to the death were classified as underlying conditions (e.g. HIV infection). The immediate cause of death and not the underlying disease was considered as the main cause of death diagnosis (e.g. disseminated histoplasmosis in an HIV-positive patient). In all cases, the assignment was made after discussion of the complete information (clinical data, images, pathology and microbiology results). In case of disagreement, three independent experts (N.R., M.J.M., J.C.H) reviewed all the data including the histological slides and provided a final diagnosis.

### Identification and characterization of *Histoplasma* infections

The presence of *Histoplasma* was screened in all 61 cases in both lungs. The screening was conducted using both histological methods (Grocott-Gomori silver stain [GMS]) and a real-time PCR. Two different real-time PCR methods were used, a SYBR green-based method with melting curve analysis and a Taqman based assay [29, 30]. In all cases with evidence of *Histoplasma* infection in lungs (either by the observation of the fungi in the histological evaluation or by a positive PCR), the liver, spleen, bone marrow, and central nervous system were tested by both GMS stain and PCR. The samples of heart, kidney, adrenal gland, lymph node, bowel, pancreas, uterus and skin were analyzed only with GMS. Finally, blood and cerebrospinal fluid were tested by PCR.

*Histoplasma* molecular typing was performed by PCR amplification and Sanger sequencing of the *tub*, *arf* and *h-anti* genes following a previously published Multilocus Sequencing Typing protocol [31]. A DNA matrix of over 250 sequences comprising the current known *Histoplasma* diversity was used for the molecular systematics studies [6]. The sequences from the current study were added to the dataset, aligned using the ClustalW matrix and finally manually inspected in the BioEdit software [32]. Maximum likelihood phylogenetic analysis was performed using the IQTREE software [33], and the best nucleotide substitution model was calculated using the–m MPF (ModelFinder) function [34]. One thousand ultrafast bootstraps and SH-like approximate likelihood ratio test were used to calculate the branch support [35]. Finally, we visualized the best Maximum likelihood tree topology using the FigTree software [36] and compared the strains identified in the study with other well-characterized *Histoplasma* species and lineages.

### Classification of *Histoplasma* infections

Three categories of *Histoplasma* infection were defined. Disseminated histoplasmosis was defined by the histological and microbiological evidence of *Histoplasma* in more than one organ or fluid. Localized pulmonary histoplasmosis was diagnosed in cases with *Histoplasma* infection detected by microbiological and histological methods, limited to the lungs. Finally, *Histoplasma* DNA detection was defined as the detection of *Histoplasma* by molecular tests in absence of histological lesions or visible fungi in the GMS stain. The inclusion of a case in this latter category required: i) a positive result by the two PCR methods, or ii) a positive result with the SYBR green-based method with confirmation of the identity of the amplicon by Sanger sequencing.

## Results

### Study cohort

During the study period, 176 deaths occurred at the hospital. An autopsy was performed approximately to one-third of the deceased patients, a percentage slightly higher than the percentage of autopsies routinely performed at the institution (18%). In the overall group of the deceased patients 65% were HIV-positive, median age was 36.3 years (range 13.9 to 82.5 years), and 126 (71%) were males.

The study included 61 patients. Fifty-nine patients were adults (two of them were maternal deaths), and two patients were children (both 13 years-old). The median age of the study group was 34.8 years (range 13.9 to 81.5 years) and 39 (64%) were males.

### Detection of *Histoplasma*

Evidence of *Histoplasma* infection was identified in 21/61 cases (34%). Disseminated histoplasmosis was diagnosed in eight cases (13%). In six of these patients (6/61, 10%) was the final cause of death. In the remaining two cases (3%), histoplasmosis was considered as a condition significantly contributing to death, but in association with another opportunistic infection (cryptococcal meningitis in one case and *Pneumocystis jirovecii* pneumonia in the second case).

Six patients (10%) had localized pulmonary histoplasmosis and seven (11%) showed *Histoplasma* DNA detection restricted to the lungs.

### Association with HIV

Thirty-seven out of the 61 (61%) patients included in the study tested positive for HIV. Fourteen out of the 21 patients with *Histoplasma* infection (67%) were HIV-positive, including all the eight patients with disseminated histoplasmosis, 4/6 with localized pulmonary histoplasmosis and 2/7 with *Histoplasma* DNA detection. Remarkably, 14 out of 37 HIV-positive patients (38%) had *Histoplasma* infection. *Histoplasma* was the main cause of death in 16% (6/37), and significantly contributed to death in 22% (8/37) of HIV-positive patients.

### Clinical characteristics of the patients with disseminated histoplasmosis

The demographic characteristics, HIV status, HIV viral load, pre-mortem CD4 cell counts, antiretroviral and antifungal treatment, clinical signs and symptoms, and the final cause of death of the eight disseminated histoplasmosis are summarized in Table 1. The median age of these patients was 39.8 years (range 27–78 years) and 7/8 cases were males. Only three patients were receiving antiretroviral therapy. The HIV infection was suspected in all eight cases during hospitalization, but could be confirmed during life only in six patients. The last CD4 count was available in six cases, with a median count of 52 cells/mL (range 16–110 cells/mL). Diarrhea was the most common symptom (six patients), followed by abdominal pain, weight loss, and dyspnea (three cases each), and fever, cough, and headache (two cases each). Upon admission to hospital, two patients were lethargic, and one showed decreased level of consciousness with focal neurological deficits. Chest radiograph was conducted in seven cases, and showed radiological lesions in five cases (reticular or interstitial pattern in three cases and consolidation in two cases). The liver transaminase levels were elevated in six patients.

**Table 1:** Demographical, clinical characteristics and antifungal treatment of the eight patients with disseminated histoplasmosis.

Case	Age	Sex	HIV status	HIV load copies/mL	Last CD4 count cells/mL	Anti-retroviral treatment	Antifungal treatment	Main symptoms and signs	Underlying condition	Immediate cause of death	Other contributing conditions or coinfections
**423**	48	M	+	4,390	110	Yes 2 months	Fluconazole	Diarrhea, weight loss, asthenia, edema, cachexia	HIV	Disseminated histoplasmosis	*E*.*Coli*, Cytomegalovirus, *T*.*gondii*, Candidiasis
**425**	32	M	+	15,400	NA	No	Amphotericin B	Dyspnea, abdominal pain, oliguria, hematuria, dysuria, skin lesions, kidney failure	HIV	Disseminated histoplasmosis	Cytomegalovirus, *T*.*gondii* Candidiasis
**428**	27	M	+	27	16	Yes 1 month	Fluconazole*	Fever, diarrhea, abdominal pain, chills, lower extremity weakness, macular skin lesions, cognitive impairment, decreased consciousness	HIV	Disseminated histoplasmosis	Cytomegalovirus Candidiasis
**457**	34	M	+	NA	NA	No	Fluconazole	Dyspnea, diarrhea, weight loss, lethargy	HIV	Disseminated histoplasmosis	Cytomegalovirus *P*. *jirovecii* *T*.*gondii* Candidiasis
**442**	78	M	+	31,500	25	No	Fluconazole	Diarrhea, cough, dysphagia	HIV	Disseminated histoplasmosis	*P*. *jirovecii*, *T*.*gondii*, *M*.*tuberculosis*
**452**	27	F	+	NA	66	NA	Fluconazole	Headache, dysphagia, weight loss, lethargy, cachexia	HIV	Disseminated histoplasmosis	Cytomegalovirus *T*.*gondii*
**460**	34	M	+	17,800	55	Yes	Fluconazole, Amphotericin B	Diarrhea, vomiting, abdominal pain	HIV	Meningitis (*Cryptococcus neoformans*)	Disseminated histoplasmosis Cytomegalovirus *T*.*gondii*
**456**	39	M	+	66,600	42	No	Fluconazole	Fever, cough, dyspnea, diarrhea, headache, thoracic pain, night sweats	HIV	Pneumonia (*Pneumocystis jirovecii*)	Disseminated histoplasmosis Cytomegalovirus

NA: not available; M: male; F: female; NA: not available data

* This patient received prophylaxis with fluconazole prior to hospital admission.

Histoplasmosis was included in the clinical differential diagnosis in two cases but was considered as the cause of death by the clinicians in only one case. In six patients, *Histoplasmosis* had not been clinically suspected; four of these cases were clinically suspicious of tuberculosis. By protocol, all eight patients received antifungal treatment during hospitalization. Six patients were treated only with fluconazole, one received both fluconazole and Amphotericin B, and one received only Amphotericin B (Table 1). A single patient received antifungal prophylaxis prior to admission to hospital (only fluconazole). All patients with disseminated histoplasmosis had other typical HIV-related opportunistic co-infections (cytomegalovirus in seven cases, toxoplasmosis in six cases, candidiasis in four cases, pneumocystosis in two cases, and tuberculosis and cryptococcosis in one case each). Most of these opportunistic co-infections were mainly diagnosed based on molecular analysis and did not have any associated histological lesions.

The mean time between admission to death was 12 days (range 0.5–50.9 days). Two patients died in the first day of admission.

### Pathological and microbiological findings of the disseminated histoplasmosis

Table 2 shows the results of the real-time PCR for *Histoplasma* and the GMS staining in disseminated histoplasmosis. Both lungs, the liver, and spleen were involved in all the eight patients. The following most affected organs were bone marrow (seven cases), central nervous system, heart, kidney, and adrenal gland (four cases each), bowel and lymph node (three cases each), and skin, pancreas and bladder (two cases each).

**Table 2:** Results of the real-time PCR for *Histoplasma* and the Grocott-Gomori metenamine silver (GMS) stain in the eight cases of disseminated histoplasmosis.

Case	Right lung PCR/GMS	Left lung PCR/GMS	Liver PCR/GMS	Spleen PCR/GMS	Bone marrow PCR/GMS	CNS PCR/GMS	Heart GMS	Kidney GMS	Adrenal gland GMS	Lymph node GMS	Bowel GMS	Skin GMS	Plasma PCR	CSF PCR
**423**	30.1 / +	33.7 / +	29.0 / +	- / +	34.0 / +	38.3 / +	+	-	+	+	-	-	-	-
**425**	20.2 / +	19.8 / +	21.9 / +	19.9 / +	NA / +	27.0 / +	+	+	+	NA	+	+	30.1	29.1
**428**	26.4 / +	25.9 / +	23.2 / +	25.5 / +	24.0 / +	28.8 / +	+	+	+	NA	-	+	32.2	35.4
**457**	23.7 /+	25.0 / +	22.4 / +	20.8 / +	21.8 / +	29.3 / +	+	+	+	+	+	-	27.0	28.9
**442**	30.2 / +	32.8 / +	36.8 / +	36.4 / +	32.7 / -	- / -	-	-	-	+	-	-	35.6	-
**452**	36.4 / +	35.7 / +	38.1 / +	35.3 / +	31.1 / -	- / -	-	-	-	-	+	-	-	-
**460**	36.7 / +	37.2 / +	- / +	36.5 / +	- / -	- / -	-	+	-	-	-	-	-	-
**456**	36.7 / +	37.2 / -	38.0 / +	36.5 / -	- / +	- / -	-	-	-	-	-	-	-	-

Cycle threshold (Ct) values of the *Histoplasma* real-time PCR assay are indicated in positive samples. Samples in which histoplasma DNA was not detected are shown as -; CNS: central nervous system; CSF: cerebrospinal fluid; NA: not available

Histologically, pulmonary lesions were ill-defined, granulomatous nodules with caseous-like necrosis, resembling typical tuberculous lesions. The lesions in other organs were more heterogeneous, including suppurative granulocytic infiltrates, disperse fungi-laden macrophages, and accumulations of visible yeast cells without presence of macrophages or any other inflammatory infiltrates. The central nervous system involvement by *Histoplasma* was very focal in the four cases and predominantly vascular, with no granulomatous reaction.

**Fig 1:**
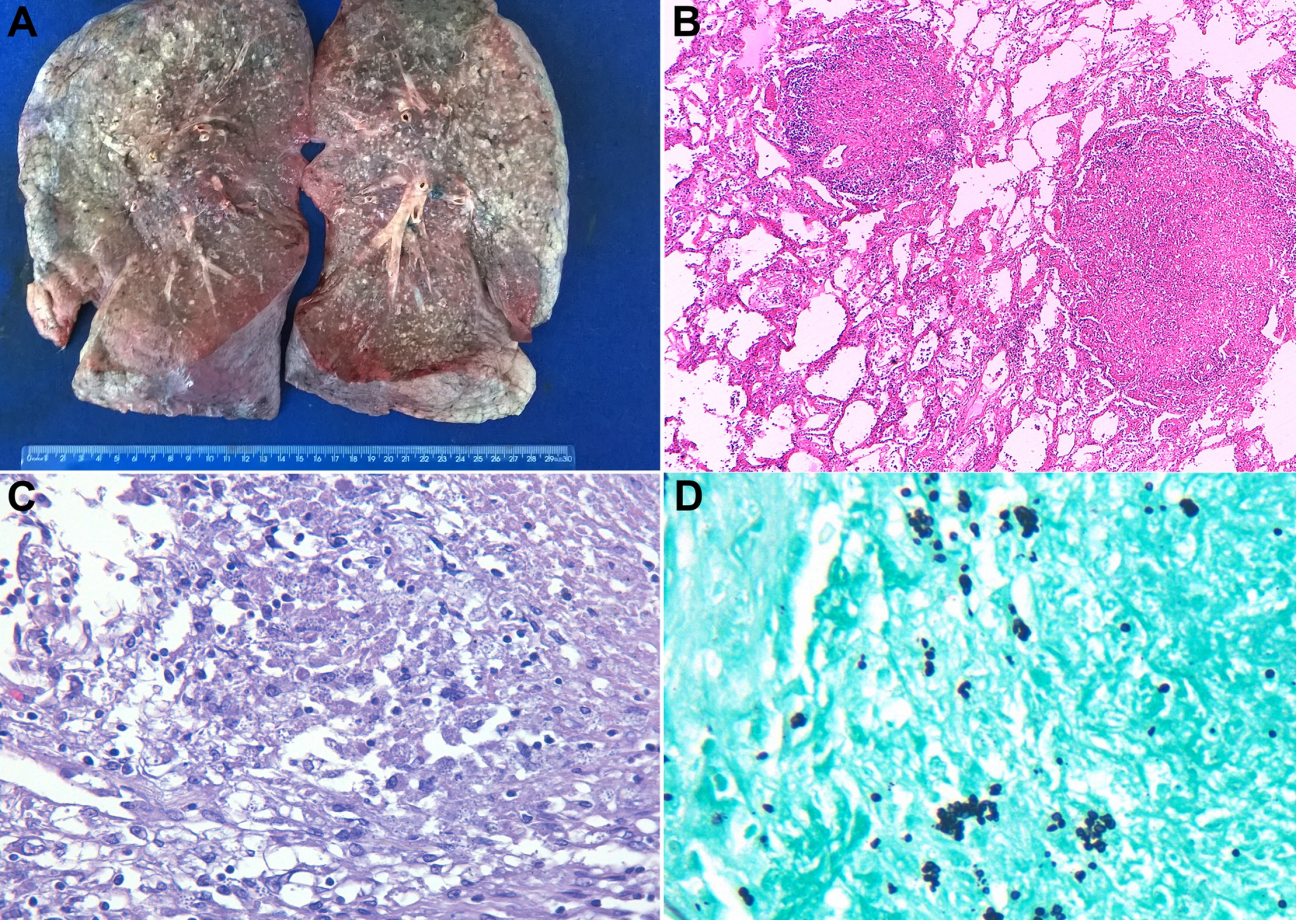
Autopsy findings in the lungs of patients with disseminated histoplasmosis. (A) Formalin-fixed lung specimen showing countless yellowish nodular lesions. (B) Lung alveoli with two ill-defined granulomatous nodules with peripheric inflammatory infiltrate simulating tuberculous lesions (hematoxylin & eosin 200x). (C) Higher magnification of lung lesions shows granulomatous infiltrate with caseous-like necrosis; fungi are visible in the cytoplasm of the macrophages (hematoxylin & eosin 400x). (D) Grocott-Gomori stain highlights small-size yeast cells, morphologically characteristic of *Histoplasma capsulatum* (Grocott-Gomori stain 600x).

**Fig 2:**
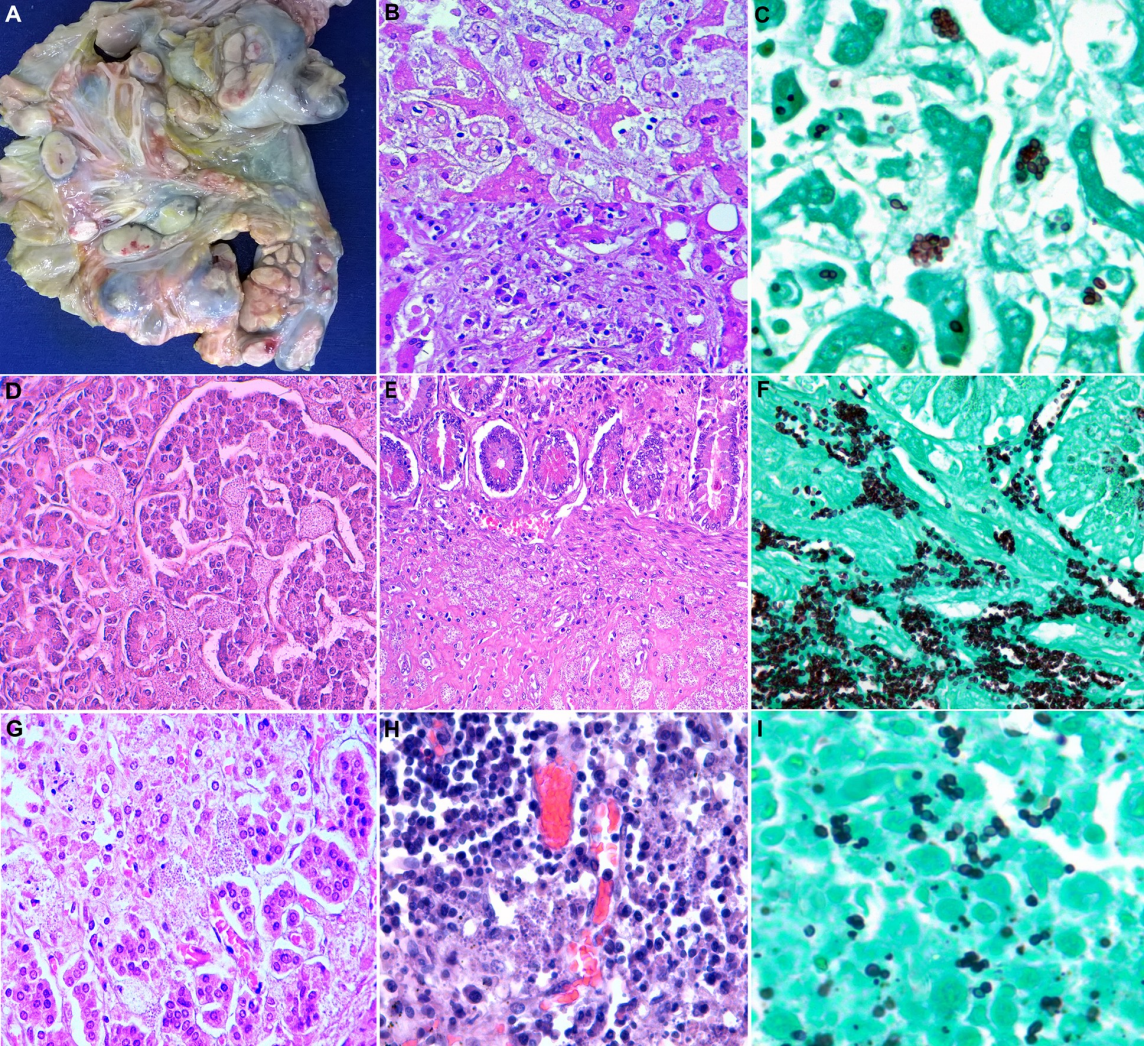
The autopsy findings in extrapulmonary organs in disseminated histoplasmosis. (A) Massive enlargement of mesenteric lymph nodes. No intestinal involvement by *Histoplasma* was observed in this case. (B) The microscopic liver lesions by *Histoplasma*: granule-laden macrophages in sinusoids and ill-defined granulomatous lesions (hematoxylin & eosin 400x). (C) Budding oval-shaped, mostly arranged in a grape cluster, *Histoplasma* yeasts positive for Grocott-Gomori stain (600x). (D) Pancreatic parenchyma showing easily identifiable, abundant fungal spores affecting acini and islet of Langerhans (hematoxylin & eosin 400x). (E) Large bowel wall with extensive submucosal involvement by visible fungal spores (hematoxylin & eosin 400x), highlighted with Grocott-Gomori stain (400x)(F). (G) and (H), Adrenal gland and lymph node, respectively, with the presence of dot-like, bluish structures, consistent with *Histoplasma* (hematoxylin & eosin 400x). (I) Isolated, paired and clustered budding of *H*. *capsulatum yeasts* stained positive with Grocott-Gomori stain (600x).

Representative images of the lung lesions and the extrapulmonary involvement are shown in Figs 1 and 2, respectively. In three patients, the fungi were identified histologically and/or microbiologically in more than ten different samples.

### Molecular typing of *Histoplasma* strains

**Fig 3:**
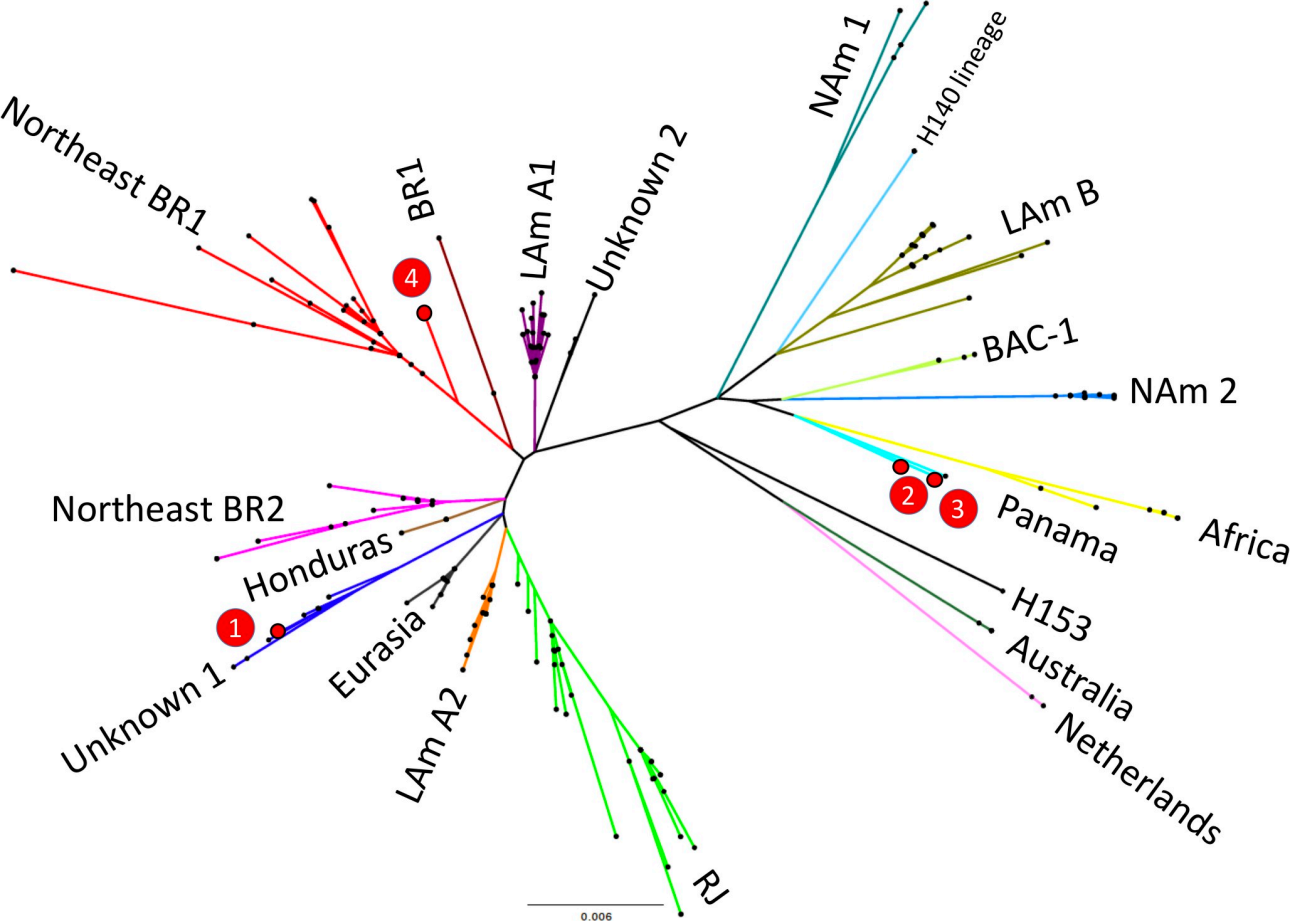
Unrooted maximum likelihood tree of 255 Histoplasma sp. taxa. Branch lengths are proportional to the number of nucleotide substitutions accumulated by every lineage (see the scale below the tree). The four genotyped strains (1 to 4) are highlighted in red and the main genetic clusters of the genus Histoplasma are shown. Nam2: North American class 2, Lam A2, LAmA1, Lam B: Latin American group A and group B, BAC: bat-associated cluster (BAC), BR: Brazil.

In four patients who died of disseminated histoplasmosis, molecular typing of *Histoplasma* was successfully achieved from DNA extracted from lung (two cases), spleen (one case) and liver (one case). Phylogenetic analysis of the sequenced strains of *Histoplasma* suggested that those belong to three different previously identified lineages as shown in Fig 3. Two strains were closely related with the Panama group whereas one strain grouped with the recently described Northeastern Brazil (BR1) clade [6] and one strain represented a yet Unknown I clade which contains other strains from Latin America.

### Characteristics of the patients with localized pulmonary histoplasmosis and those with *Histoplasma* DNA detection

Table 3 shows the main findings in six patients with localized pulmonary histoplasmosis and in seven patients with *Histoplasma* DNA detection in lungs, including the demographic characteristics, HIV status and viral load, pre-mortem CD4 count, antiretroviral and antifungal treatment received, as well as the final cause of death and the results of *Histoplasma* testing in lung samples. No evidence of *Histoplasma* was found in any other organ. In all the 13 cases, another disease was identified as the main cause of death. The six HIV-positive patients in this group died of an AIDS-associated infection. One HIV-negative individual with *Histoplasma* DNA detection was immunosuppressed due to corticosteroid treatment and received Amphotericin B due to a clinical diagnosis of oral histoplasmosis.

**Table 3:** Demographical, clinical characteristics, prophylactic treatment, results of the real-time PCR for Histoplasma and the Grocott-Gomori metenamine silver (GMS) stain in the lungs of patients with localized pulmonary histoplasmosis (LPH) and patients with Histoplasma DNA detection (HDD) in the lungs without histological lesions.

Case	Age	Sex	Type of infection	Right lung PCR/GMS	Left lung PCR/GMS	HIV	HIV load copies/mL	CD4 count cells/mL	Anti-retroviral treatment	Antifungal treatment	Underlying condition	Immediate cause of death	Other contributing conditions or coinfections
**459**	30	M	LPH	36.8 / +	- / +	+	737	6	No	Fluconazole	HIV	Disseminated cytomegalovirus	None
**435**	39	F	LPH	35.0 / +	36.4 / -	+	38,250	20	Yes	Fluconazole	HIV	Miliary tuberculosis	None
**446**	46	M	LPH	37.1 / +	37.0 / -	+	1110	187	NA	-	HIV	Cerebral toxoplasmosis	Hypertension
**427**	22	F	LPH	37.1 / +	- / -	+	540,000	NA	No	Fluconazole	HIV	Disseminated cytomegalovirus	*P*.*jirovecii*, *T*.*gondii*
**414**	72	F	LPH	36.2 / +	- / +	-	NT	NA	NA	-	Cirrhosis	Pneumonia (no agent).	Panhypopituitarism
**437**	41	F	LPH	37.1 / -	- / +	-	NT	NA	NA	-	Viral hepatitis (HBV+HDV)	Cholangiocarcinoma	Cirrhosis
**461**	50	M	HDD	37.8 / -	36.6 / -	+	226	11	Yes	-	HIV	Cytomegalovirus pneumonia	*K*. *pneumoniae*
**406**	29	M	HDD	- / -	37.8 / -	+	28,600	NA	No	Fluconazole	HIV	Miliary tuberculosis	*T*.*gondii*
**429**	39	M	HDD	37.3 / -	37.2 / -	-	NT	NA	NA	-	Viral hepatitis (HBV+HDV)	Liver failure.	Cirrhosis
**450**	47	M	HDD	36.2 / -	36.9 / -	-	NT	NA	NA	-	HTLV 1–2	Meningitis (*Neisseria meningitidis*).	*S*.*pneumoniae*
**443**	81	F	HDD	36.7 / -	36.0 / -	-	NT	NA	NA	-	None	Disseminated gastric adenocarcinoma	None
**405**	38	M	HDD	- / -	35.0 / -	-	NT	NA	NA	-	None	Sepsis (*Neisseria meningitidis*)	Adenovirus
**415**	18	F	HDD	35.6 / -	- / -	-	NT	NA	NA	Amphotericin B	Viral hepatitis (HBV)	Liver failure	None

HBV: hepatitis B virus; HDV: hepatitis D virus; HTLV: human T-lymphotrophic virus; M: male; F: female; NA: not available; NT: not tested due to insufficient amount of sample.

## Discussion

In this series of 61 complete autopsies conducted in the Brazilian Amazon, histoplasmosis was the main cause of death in 10% of the patients. In addition, *Histoplasma* significantly contributed to death in 3% of the patients, caused pulmonary disease in 10% and was detected by molecular methods in 11% of the individuals included in this series. Although the number of conducted autopsies was relatively low, the overall prevalence and mortality (34% and 13%, respectively) of *Histoplasma* infection found in our study were remarkably high. Among HIV-positive patients, the prevalence and mortality were even higher (38% and 22%, respectively). Another autopsy study conducted in Brazil reported even higher prevalence and mortality of histoplasmosis among HIV positive patients (55% and 45%, respectively) who died in the period 2005–2018 [16]. Contrarily, an autopsy-based study conducted among HIV-positive individuals between 1996–2003 in our institution found that *Histoplasma* caused 13% of deaths [37]. The higher frequency in our study might be partially explained by the thorough molecular and histological screening for *Histoplasma*. The low levels of CD4 cells and the high HIV-1 viral load observed in our cases, and in other autopsy cohort [16] highlight the elevated fatality rate of histoplasmosis in patients with advanced HIV disease in the endemic areas in Brazil. The advanced stages of HIV disease are likely the consequence of the lack of antiretroviral treatment in these patients [16, 38]. These data are striking, as this therapy is freely and widely available to all HIV positive individuals since 1997. An acceleration of anti-retroviral roll-out is thus an urgent action that would probably reduce mortality associated with histoplasmosis and with other AIDS-associated infections.

Histoplasmosis is considered a neglected disease and, although it is known to be an important contributor to HIV-related mortality in endemic areas of Latin America, many cases are clinically missed. In our study, histoplasmosis was suspected only in two out of eight patients with disseminated disease. Similar rates of underdiagnosis were reported in Brazilian autopsy cohort [16]. The non-specific clinical presentation of the disease and the fact that the microbiological diagnosis remains challenging in resource-limited areas make this infection markedly underdiagnosed [39, 40]. The definitive diagnosis of histoplasmosis has traditionally relied on culture or direct examination of clinical specimens with special stains [41]. However, these procedures are time-consuming and have low sensitivity. Molecular techniques based on PCR offer higher sensitivity [1] but are not yet fully standardized and are not routinely available in many countries where the disease is prevalent. Histological examination is particularly useful for the diagnosis of histoplasmosis [41]. However, the identification of the fungus in tissues with H&E may be challenging, especially when present in small numbers. Special stains such as GMS are particularly helpful in these cases [21], as shown in our study. Damasceno-Escoura et al [16] reported even higher diagnostic rates of disease establishing the diagnosis exclusively on histology. In recent years, novel diagnostic approaches including rapid tests have been developed [42]. Antigen detection assays in urine samples are relatively easy to perform and offer good sensitivity and specificity but still remain unavailable in most laboratories in endemic areas. Of note, newly developed ELISA tests for the detection of *Histoplasma* antigens represent a promising tool to expand the microbiological diagnosis of the disease in areas where it is mostly needed [43, 44].

The evolution of disseminated histoplasmosis is rapid and always fatal if untreated [8]. In addition to laboratory diagnostics, access to adequate treatment, which includes liposomal Amphotericin B followed by oral itraconazole for up to one year is also urgently required. Itraconazole is also recommended as prophylaxis in immunosuppressed patients [21]. In line with the high underdiagnosis rates, prophylactic or treatment strategies for histoplasmosis were clearly suboptimal in our series, simillarly to the results of a recent Brazilian series [16]. A spectrum of *Histoplasma* infections was observed in our study. Some patients had *Histoplasma* DNA detection in lungs in the absence of identifiable yeasts, while other cases showed localized pulmonary histoplasmosis with clearly identifiable yeasts in the lungs associated to mild histological lesions. The patients with only molecular evidence of *Histoplasma* likely represent initial stages of the infection in which histological lesions are either not yet developed or are restricted to lung areas that were not sampled for histological analysis. Finally, some patients had severe disseminated infections involving multiple organs, such as the lungs, spleen, and liver, in agreement with cases reported in other series [14, 15, 44]. Curiously, a recent study in a different area of Brazil [16] showed that *Histoplasma* was more frequently identified in the lymph nodes and less commonly involved the liver and the spleen compared with our series.

Interestingly, although only four strains could be subjected to molecular typing, we identified three different *Histoplasma* genotypes, consistent with a high diversity of this fungus circulating in the area of study, in line with previous reports from Latin America [45]. Two of these clades have been recently described in molecular epidemiology studies in Northeast Brazil and Colombia/Panama [46]. In our recent post-mortem study focused on cryptococcal disease-related mortality in Brazil and Mozambique, we also identified different molecular fungal types within a small sample [47]. These data highlight the usefulness of molecular typing techniques for a better characterization of fatal fungal infections.

One of the main strengths of the study is the use of the complete autopsy, the gold standard for cause of death investigation. There are also significant limitations. Firstly, it was carried out in a single tertiary hospital, specifically focused on infectious diseases. Consequently, there was a high HIV prevalence among patients and thus, the cohort might not be representative of the overall population. This could limit the extrapolation of the results to other areas or health facilities with less prevalence of HIV. Secondly, the number of severe histoplasmosis cases is relatively small.

In conclusion, our study shows the high incidence and mortality associated with disseminated histoplasmosis among HIV-positive in the Brazilian Amazon. The low index of clinical suspicion, and the severity of the disseminated disease warrant the need of implementation of sensitive routine screening methods. Moreover, antifungal prophylaxis should be encouraged in patients with advanced HIV disease living in hyper-endemic areas of histoplasmosis. Finally, upscaling of antiretroviral therapy is needed as fatal histoplasmosis occurs among patients with HIV with advanced disease who not receiving proper medical care.
